# *Weissella confusa* alleviates experimental colitis in mice by regulating inflammatory pathways and gut microbiota

**DOI:** 10.3389/fmicb.2025.1574548

**Published:** 2025-04-28

**Authors:** Huijing Tang, Qianhua Fan, Yao Lu, Xiaoying Lin, Ruiting Lan, Dalong Hu, Shuwei Zhang, Ruoshi Wang, Ruiqing Zhao, Liyun Liu, Jianguo Xu

**Affiliations:** ^1^Department of Epidemiology, Center for Global Health, School of Public Health, Nanjing Medical University, Nanjing, China; ^2^National Key Laboratory of Intelligent Tracking and Forecasting for Infectious Diseases, National Institute for Communicable Disease Control and Prevention, Chinese Center for Disease Control and Prevention, Beijing, China; ^3^School of Biotechnology and Biomolecular Sciences, University of New South Wales, Sydney, NSW, Australia; ^4^Research Units of Discovery of Unknown Bacteria and Function, Chinese Academy of Medical Sciences, Beijing, China; ^5^Hebei Key Laboratory of Intractable Pathogens, Shijiazhuang Center for Disease Control and Prevention, Shijiazhuang, China

**Keywords:** *Weissella confusa*, colitis, gut microbiota, cytokine, anti-inflammatory

## Abstract

**Background:**

Inflammatory bowel disease (IBD) is a chronic condition with no cure. Probiotics may offer a new strategy for the treatment of IBD. *Weissella confusa* has been shown to have antibacterial, anti-inflammatory, and antioxidant beneficial effects in animal models. However, the anti-inflammatory effect of *W. confusa* at host cellular level and their effect on the gut microbiota are unclear. This study aimed to investigate the effects of *W. confusa* Wc1982 on inflammation and gut microbiota alterations in a dextran sulfate sodium (DSS) induced colitis mouse model.

**Method:**

Female C57BL/6J mice were randomly divided into control, DSS, and Wc1982 groups (*n* = 6/group). The Wc1982 group was given continuous gavage of *W. confusa* Wc1982 for 14 days with the last 7 days also treated with 3% DSS. Disease phenotypes including daily body weight, disease activity index (DAI), colon length and histological changes were evaluated. The composition of colon flora, α-diversity and β-diversity were analyzed by 16S rRNA sequencing. The colonic gene expression profile was analyzed by RNA-seq, and serum and colonic proinflammatory cytokines were assessed by enzyme-linked immunosorbent assay. Analysis of variance (ANOVA) was used to analyze the differences among groups, and Spearman rank test was used to calculate the correlation between species relative abundance and pro-inflammatory markers.

**Results:**

Compared with DSS group, *W. confusa* Wc1982 significantly improved the disease phenotypes of colitis mice including decreased DAI and pathological score and reduced colon shortening, decreased colonic IL-17, IL-6, and TNF-α levels and serum lipopolysaccharide (*p* < 0.05), and downregulated the expression of key genes of IL-17 pathway (*Lcn2*, *Mmp3*, *Mmp13*, *Ptgs2*; *p* < 0.05). *W. confusa* Wc1982 modified the gut microbiota community of colitis mice, with increased α-diversity, increased abundance of *W. confusa* and *Akkermansia muciniphila*, and decreased abundance of *Enterococcus faecalis* and *Escherichia coli* (all *p* < 0.05).

**Conclusion:**

Supplementation with *W. confusa* Wc1982 offers a promising strategy for alleviating colitis.

## 1 Introduction

Inflammatory bowel disease (IBD) is a chronic condition characterized by redness, swelling, inflammatory infiltrate, and ulcer formation in the intestinal mucosa (Peyrin-Biroulet et al., 2016). The occurrence and development of IBD are related to many factors, such as genetics, abnormal immune response, intestinal barrier disorders, and gastrointestinal microbiota disorders (Ye et al., 2015; Guan, 2019). Gut microbiota dysbiosis, especially the decline in the abundance and diversity of specific genera, is an important factor in the induction of IBD (Haneishi et al., 2023). It has been reported that in the fecal microbiota of IBD patients, Proteobacteria dominated by *Escherichia coli* was relatively increased (Nishida et al., 2018), while *Akkermansia muciniphila* decreased (Wang et al., 2020). Other studies have shown that adherent-invasive *E. coli* disrupts the integrity of the epithelial barrier by invading intestinal epithelial cells and triggering mitochondrial destruction, which increases inflammation and accelerates the progression of colitis (Hamed et al., 2023), whereas *A. muciniphila* degrades mucins to produce acetic acid, activates adenosine 5′-monophosphate-activated protein kinase pathway, up-regulates tight junction proteins (ZO-1, occludin), thereby enhancing intestinal barrier and reducing pro-inflammatory factor (TNF-α, IL-6) secretion (Qian et al., 2022).

Probiotics, as living microorganisms, have a positive effect on the gut by regulating the immune response, increasing the production of mucosal IgA, and competing with pathological bacteria, which has become a new strategy for the treatment of IBD (Vallejos et al., 2025; Selvamani et al., 2022; Mei et al., 2019; Martyniak et al., 2021; Goodoory et al., 2023). *Lactobacillus* and *Bifidobacterium* have been well studied as probiotics for IBD intervention due to their well-characterized immunomodulatory effect, such as NF-κB pathway inhibition and IL-10 induction (Wong et al., 2022; Wang et al., 2023; Haque et al., 2024). *Weissella confusa* is a Gram-positive heterofermentative lactic acid bacteria, widely distributed in saliva, breast milk, human gastrointestinal tract and traditional fermented foods, with anti-inflammatory, antioxidant and antibacterial activities comparable to traditional probiotics (Liu et al., 2023; Tian et al., 2025) and thus has the potential as a new probiotic. *W. confusa* strain DD_A7 has exhibited significant anti-inflammatory effects by reducing oxidative stress and modulating the NF-κB signaling pathway (Dey and Kang, 2020). In RAW 264.7 cells activated by *Escherichia coli*, *W. confusa* DD_A7 downregulated the expression of the iNOS gene, which regulated the production of nitric oxide, a pro-inflammatory mediator produced by L-arginine (Dey et al., 2019). When co-cultured with *Pseudomonas nitroreducens*, *W. confusa* strain NRRL-B-14171, significantly reduced the secretion of the anti-inflammatory cytokine IL-25 in HT-29 and Huh7 cells (Ghadimi et al., 2023). *W. confusa* strain F213 was tested in a rat model of colitis induced by dextran sulfate sodium (DSS) (Sujaya et al., 2023). Supplementation of *W. confusa* F213 significantly alleviated DSS induced body weight loss, reduced inflammatory cell infiltration, and significantly upregulated the expression of tight junction protein ZO-1 (Sujaya et al., 2023). However, these studies did not investigate changes in host transcriptome and gut microbiome structure after intervention.

In view of this knowledge gap, this study established a dextran sulfate sodium (DSS) induced colitis mouse model, and evaluated the intervention effect of *W. confusa* strain Wc1982 isolated from feces of a healthy donor by using a multiomic strategy of “transcriptome-microbiome.” It was revealed that *W. confusa* Wc1982 alleviated colitis symptoms by regulating the expression of key genes of IL-17 signaling pathway (*Lcn2*, *Mmp3*, *Mmp13*, *Ptgs2*) and reshaping the structure of intestinal flora (increasing α-diversity, *W. confusa* and *Akkermansia muciniphila*, decreasing *Enterococcus faecalis* and *Escherichia coli*).

## 2 Materials and methods

### 2.1 Bacterial strains and culture conditions

*W. confusa* Wc1982 was isolated from a fecal sample of a healthy individual, followed by 16S rRNA gene sequencing and species identification using BLAST in the EzBioCloud database. *W. confusa* Wc1982 has been stored in the China General Microbiological Culture Collection Center (CGMCC) with preservation number CGMCC no. 27139. *W. confusa* Wc1982 was sub-cultured on de Man, Rogosa and Sharpe (MRS) agar (Land Bridge Technology Co., Ltd., Beijing, China) at 37°C for 24 h. For the oral treatment of mice, the viable count was adjusted to 5 × 10^8^ CFU/mL using phosphate-buffered saline (PBS).

### 2.2 Construction of colitis model and experimental design

**Figure 1:**
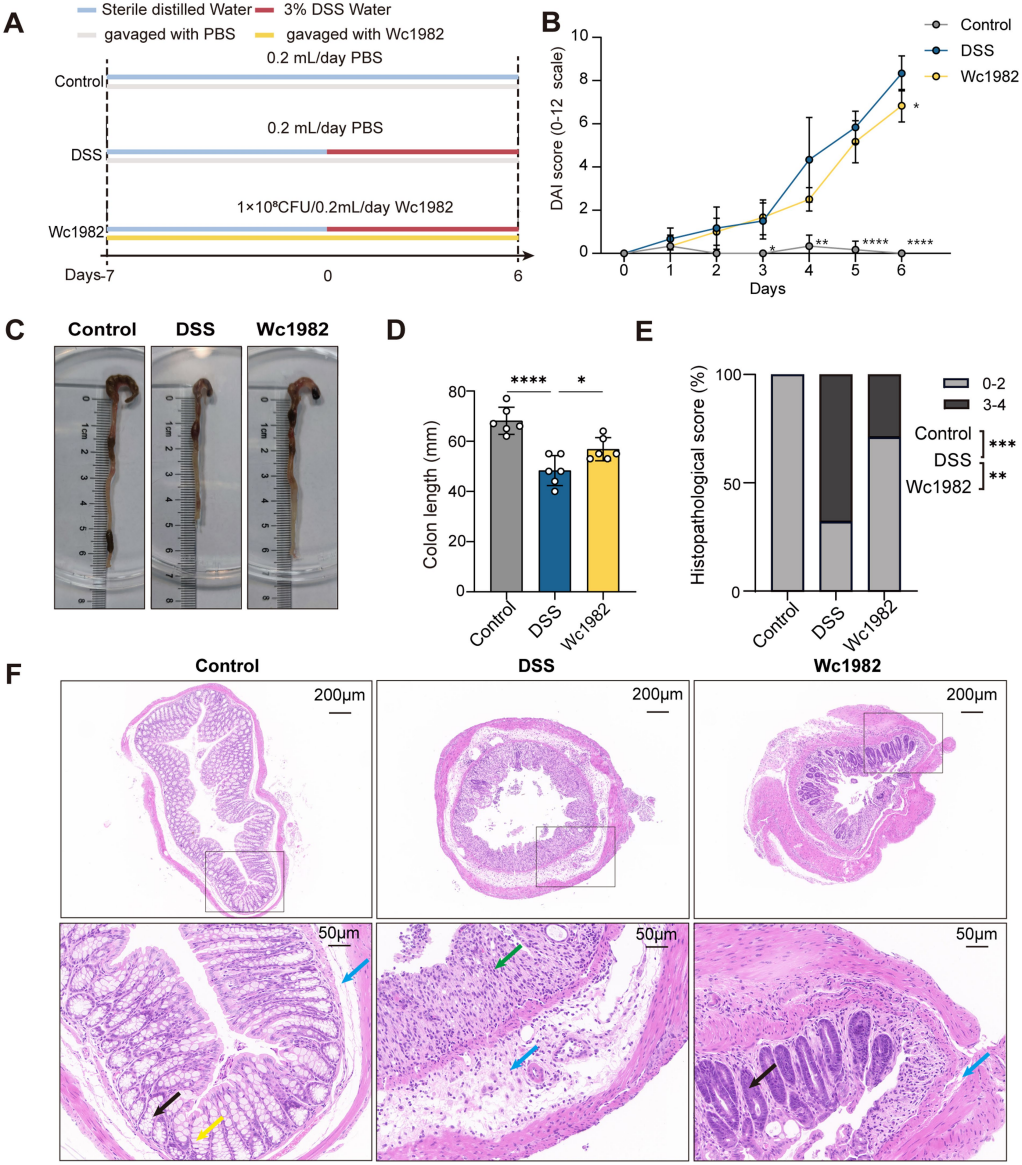
Effects of *W. confusa* Wc1982 on the DSS-induced colitis in C57BL/6J mice. **(A)** Schematic representation of the experimental design, no of mice per group *n* = 6. **(B)** DAI score of mice in each group from day 0 to 6. **(C)** Representative images of the colons. **(D)** The colon lengths of mice in each group. **(E)** Distribution of histopathological scores. **(F)** Representative images of H&E-stained sections of colons from indicated groups; crypts (black arrow); inflammatory cell infiltration (green arrow); submucosal layer (blue arrow); goblet cell (yellow arrow). Data presented as mean ± standard deviation (SD). Statistical comparison was performed by two-way ANOVA followed by Tukey’s multiple comparison test **(B)**, one-way ANOVA followed by Holm–Šidák’s multiple comparison test **(D)** or Fisher’s exact test **(E)**. Comparisons were made between control vs. DSS and Wc1982 vs. DSS groups. ^*^p < 0.05, ^**^p < 0.01, ^***^p < 0.001, and ^****^p < 0.0001.

The animal experiment was approved by the Welfare & Ethical Inspection in Animal Experimentation Committee of the Chinese CDC (Approval No. 2023-032). Specific-pathogen-free (SPF) female C57BL/6 J mice (6 weeks old) weighing 16–18 g were purchased from Beijing Vital River Laboratory Animal Technology. After a 3-day acclimation, mice were randomly assigned to one of the groups with six mice per group: control, DSS, and Wc1982 groups using the blockrand package (version 1.5) in Rstudio: (1) control: the control group (oral gavage with 0.2 mL PBS/day), (2) DSS: PBS treatment group (oral gavage with 0.2 mL PBS/day), and (3) Wc1982: *W. confusa* Wc1982 treatment group (oral gavage with 0.2 mL bacterial suspension/day). All groups were provided with free access to sterile distilled water and food. As shown in Figure 1A, Wc1982 intervention lasted from day −7 to day 6, while colitis was induced in the DSS and Wc1982 groups by administering 3.0% DSS in their drinking water from day 0 to day 6. Wc1982 was administered daily from day −7 to day 6, covering both the pre-DSS preventive phase and the DSS induction period, with no further treatment after DSS cessation. From day 0 to day 6, fecal properties and occult blood status were recorded daily. The disease activity index (DAI) score, which assesses the degree and severity of IBD as described in Supplementary Table S1 (Sann et al., 2013; Li Q. et al., 2022), was calculated in days 0–6. Euthanasia was performed using a graded CO_2_ inhalation system (30% chamber displacement rate) followed by cervical dislocation to ensure death. All procedures were conducted in strict accordance with the 2020 AVMA Guidelines for the Euthanasia of Animals. After euthanasia, whole blood from the mice was collected and centrifuged at 3,000 rpm for 10 min to obtain serum. The lengths of the colon from the ileocecal junction to the anus were measured. The colons were collected for histopathological observation and transcriptome analysis, and the cecum contents were collected for microflora analysis.

### 2.3 Histopathological analysis

The distal colon segment of each mouse was embedded in paraffin and followed by hematoxylin-eosin (H&E) staining. The degree of inflammation and histopathological injury lesions of the colon were analyzed with reference (Supplementary Table S2) (Wang et al., 2017).

### 2.4 Colon transcriptome analysis

Total RNA was isolated from colonic tissue using TRIzol reagent (Life Technologies). The quality and integrity of the RNA were assessed with the RNA Nano 2000 Assay Kit on the Agilent Bioanalyzer 2100 system (Agilent Technologies) (Walker et al., 2023). The library construction process involved mRNA enrichment, fragmentation, cDNA synthesis and purification, end repair with adapter ligation, size selection, and PCR enrichment to create a cDNA library. Initial quantification was performed using the Qubit 3.0 Fluorometer, with the concentration required to be above 1 ng/μL. Subsequently, the Qsep400 high-throughput analysis system was used to detect the insert fragments of the library. The effective concentration of the library (library effective concentration >2 nM) was accurately quantified using the quantitative polymerase chain reaction to ensure library quality. After the library passes quality control, PE150 mode sequencing was performed using a high-throughput sequencing platform. Differentially expressed genes (DEGs) were identified using the EBSeq 3.18 package with a threshold of *p* < 0.05 and fold-change >1.5 (Li D. et al., 2022; Li Q. et al., 2022). To elucidate the biological roles of colonic DEGs, we performed a comprehensive pathway analysis using the Kyoto Encyclopedia of Genes and Genomes (KEGG) database. Gene set enrichment analysis (GSEA) was applied to pre-ranked colon genes based on the log2 transformed fold change in expression. Gene sets with a false discovery rate *q* < 0.05 and an absolute value of normalized enrichment score (|NES|) >1.0 were considered statistically significant. The RNA-seq data is publicly available in NCBI under the accession number PRJNA1201878.

### 2.5 Real-time quantitative PCR

The total RNA was isolated from colon tissues using TRIzol Reagent (Invitrogen, Carlsbad, CA, United States). The cDNA was synthesized from the total RNA using the PrimeScript^™^ RT Reagent Kit (Perfect Real Time, TaKaRa). The real-time quantitative PCR (qRT-PCR) was performed using SYBR Premix Ex Taq II (Perfect Real Time; TaKaRa) on the Rotor-Gene Q thermal cycler system (Qiagen, Valencia, CA) under the following conditions: initial heat activation at 95°C for 1 min, denaturation at 95°C for 30 s, annealing at 60°C for 30 s, and extension at 72°C for 45 s (40 cycles). *Gapdh* was used as the internal reference, and the $2^{-\mathrm{\Delta \Delta CT}}$ method was used to calculate the expression levels of the related genes. All primer sequences are shown in Supplementary Table S3.

### 2.6 Biochemical analysis

Levels of IL-17, IL-6, and TNF-α in serum and colonic tissue were detected by enzyme-linked immunosorbent assay (R&D Systems, Minneapolis, MN, United States) according to the manufacturer’s instructions. Serum lipopolysaccharide (LPS) was detected by a commercial diagnostic kit (Beijing Solarbio Science & Technology Co., Ltd., China).

### 2.7 Microbiota 16S rRNA gene sequencing

Cecum contents DNA were extracted using the TGuide S96 Magnetic Soil/Stool DNA Kit (Tiangen Biotech (Beijing) Co., Ltd.), and the concentration and integrity of the DNA samples were assessed. Genomic DNA served as a template for amplifying the full-length bacterial 16S rRNA gene using universal primers 27F (5′-AGRGTTYGATYMTGGCTCAG-3′) and 1492R (5′-RGYTACCT TGTTACGACTT-3′) targeting the V1–V9 hypervariable regions (Klindworth et al., 2013). The total PCR amplicons were purified with VAHTS^™^ DNA Clean Beads (Vazyme, Nanjing, China) and quantified using the Qubit dsDNA HS Assay Kit and Qubit 3.0 Fluorometer (Invitrogen, Thermo Fisher Scientific, Oregon, United States). The PCR amplicons were sequenced on the Pacific Biosciences SMRT RS II platform, a third-generation sequencing system employing Single Molecule Real-Time (SMRT) technology (Moraes et al., 2020). SMRTbell libraries were prepared from the amplified DNA by SMRTbell Express Template Prep Kit 2.0 according to the manufacturer’s instructions provided by Pacific Biosciences. Circular consensus sequences were extracted from raw data followed by barcode recognition, length filtering, and chimera removal via UCHIME algorithm (Li et al., 2014; Edgar et al., 2011). High-quality sequences were clustered into Operational Taxonomic Units (OTUs) at 97% similarity using the VSEARCH algorithm (Rognes et al., 2016). Taxonomic classification was performed against the SILVA 138.1 reference database with a confidence threshold of 80% (Quast et al., 2013).

The microbial compositional profiling (barplot), LEfSe analysis, α-diversity indices (Shannon, Simpson, Chao1, ACE), and β-diversity were analyzed using the BMK Cloud Platform and QIIME2 2022.2 pipeline (Bokulich et al., 2018). Results were visualized with GraphPad Prism 9.0. The microbiota 16S rRNA gene sequencing data is publicly available in NCBI under the accession number PRJNA1178434.

### 2.8 Statistical analysis

Results were predominantly shown as mean ± Standard Deviation (SD). Statistical analysis and graphics were performed by GraphPad Prism 9.0 and R 4.4.0. Differences between groups were analyzed by analysis of variance (one-way ANOVA or two-way ANOVA). The Kruskal–Wallis test was performed on nonparametric variables. Correlations between changes in species relative abundance and proinflammatory markers were calculated by Spearman’s rank test. Statistical significance is indicated as follows: ^*^p < 0.05, ^**^p < 0.01, ^***^p < 0.001, and ^****^p < 0.0001 and NS means no significance.

## 3 Results

### 3.1 *W. confusa* Wc1982 alleviated DSS-induced colitis in mice

To investigate the anti-inflammatory effect of *W. confusa*, Wc1982 was administered to mice with DSS-induced colitis (Figure 1A). There were three experimental groups of mice with the DSS induction + PBS, DSS induction + Wc1982 intervention, and PBS treatment only (no DSS induction or Wc1982 intervention), referred to as the DSS group, Wc1982 group, and control group, respectively. Weight changes were tracked daily. During the induction of DSS, compared with the control group, the DSS group displayed notable weight loss on days 5 and 6 (*p* < 0.05, Supplementary Figure S1), however, the weight losses were comparable between the DSS and Wc1982 groups (*p* < 0.05, Supplementary Figure S1). On days 3–6 of DSS exposure, the DAI score of the DSS group was significantly higher than that of the control group, and the DAI score of the Wc1982 group was significantly lower than that of the DSS group on day 6 (*p* < 0.05, Figure 1B). Compared with the control group, the colon length was significantly shortened in the DSS group, and the colon shortening was significantly less in the Wc1982 group than in the DSS group (*p* < 0.05, Figures 1C,D).

As shown in Figure 1E, histopathological scores were higher in the DSS group than in the control group (*p* < 0.001, Fisher’s exact test) and the Wc1982 group (*p* < 0.01, Fisher’s exact test). Histopathological examination revealed significant intestinal inflammation in the DSS group, characterized by loss of goblet cells, thickened mucosa, submucosal edema, crypt atrophy, inflammatory cell infiltration, and tissue proliferation. In contrast, the Wc1982 group showed milder pathological changes, including reduced epithelial detachment and inflammatory cell infiltration, improved crypt structure preservation, and reduced epithelial erosion. The control group displayed normal intestinal histology with no signs of inflammation or tissue damage (Figure 1F).

### 3.2 Effects of *W. confusa* Wc1982 on colonic gene expression in DSS-induced colitis mice

**Figure 2:**
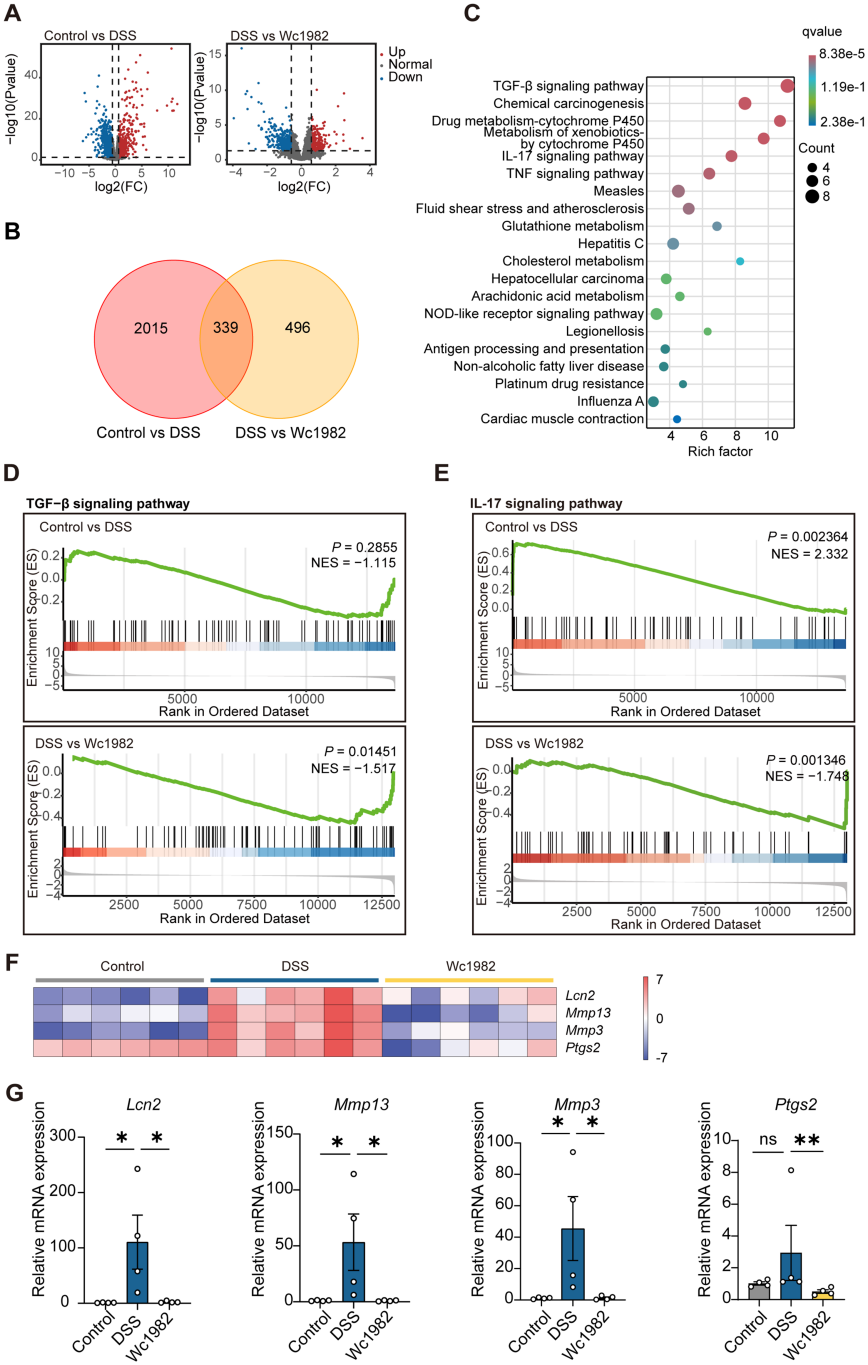
Transcriptomic analysis of colon tissues. **(A)** Volcano plot of DEGs. **(B)** Venn diagram displaying the overlapping genes. **(C)** KEGG pathway enrichment analysis of 76 inflammation-related overlapping genes. **(D,E)** GSEA of the TGF-β and IL-17 signaling pathway gene set (|NES| >1, *p* < 0.05). **(F)** The heatmap displaying the expression profile of core DEGs in the IL-17 signaling pathway. **(G)** The mRNA levels of *Lcn2*, *Mmp13*, *Mmp3*, and *Ptgs2* in colon tissues. NES, normalized enrichment score. Data presented as mean ± standard deviation (SD). Statistical comparison was performed by Kruskal–Wallis test with Dunn’s multiple comparison test. ^*^p < 0.05 and ^**^p < 0.01.

To investigate the molecular mechanisms by which *W. confusa* Wc1982 affects colon inflammation, we performed RNA sequencing on colon tissues to determine differential gene expression profiles of the different treatment groups. Compared with the control group, the DSS group exhibited 942 upregulated and 1,412 downregulated genes, while the Wc1982 group showed 268 upregulated and 567 downregulated genes compared to the DSS group (Figure 2A, *p* < 0.05, fold change >1.5). The Venn diagram displayed 339 overlapping genes among groups of control vs. DSS and DSS vs. Wc1982 (Figure 2B).

KEGG enrichment analysis showed that 76 overlapping inflammation-related genes were mainly involved in the TGF-β signaling pathway and IL-17 signaling pathway (Supplementary Table S4 and Figure 2C). Further GSEA analysis of mouse colon transcripts showed that the IL-17 signaling pathway was up-regulated in the DSS group compared with the control group, while the TGF-β signaling pathway was comparable; the IL-17 signaling pathway and TGF-β signaling pathway were down-regulated in the Wc1982 group compared with the DSS group (Figures 2D,E). Subsequently, the heatmap showed the four core DEGs, *Lcn2*, *Mmp13*, *Mmp3*, and *Ptgs2*, in the IL-17 signaling pathway (Figure 2F), which were confirmed by qRT-PCR, and the results showed that all four genes exhibited a concordant direction in both RNA-Seq and qRT-PCR (*p* < 0.05, Figure 2G).

### 3.3 Effects of *W. confusa* Wc1982 on cytokines and LPS

**Figure 3:**
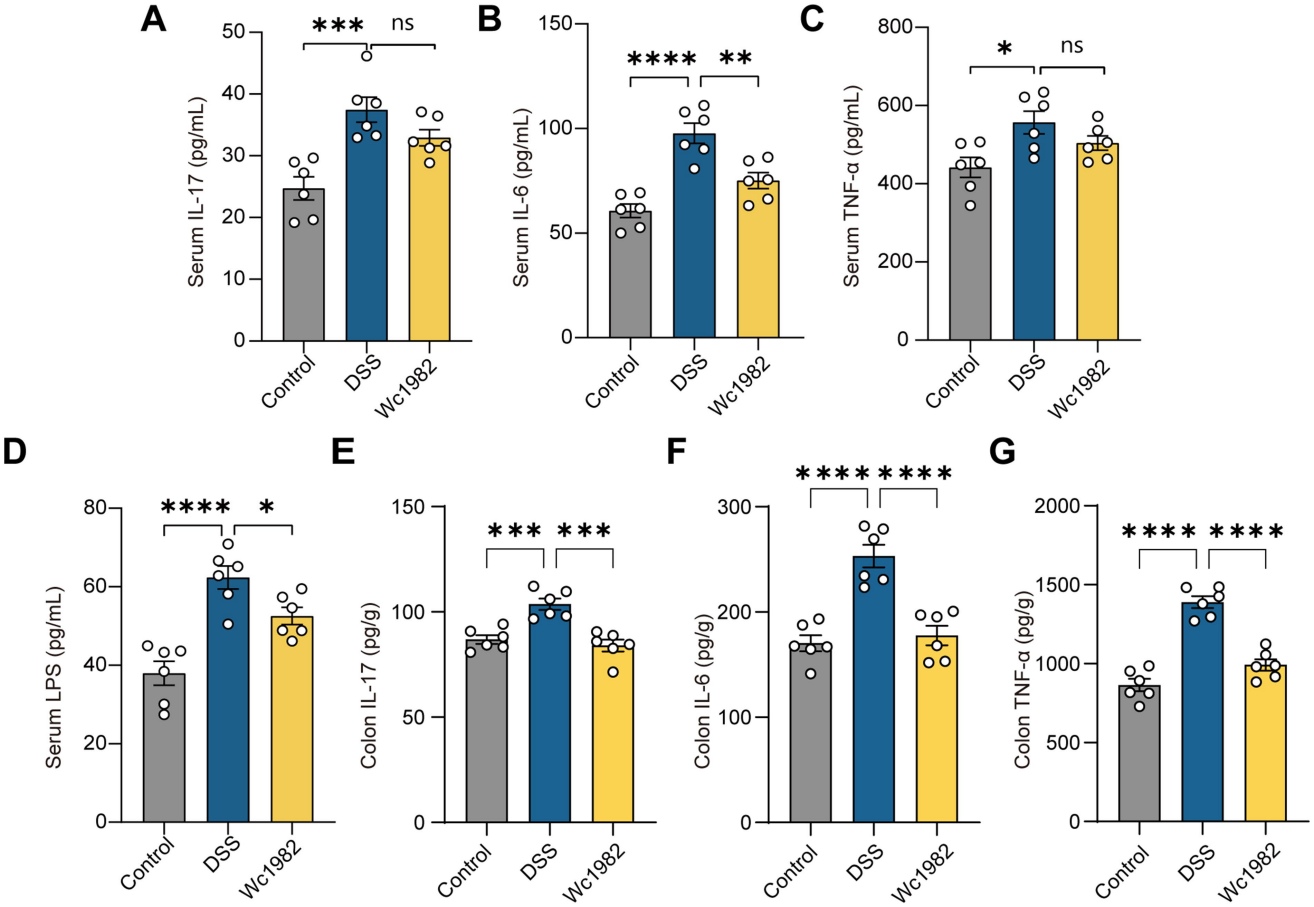
Effects of *W. confusa* Wc1982 on cytokines and LPS. **(A–D)** Levels of IL-17, IL-6, TNF-α, and LPS in mice serum. **(E–G)** Levels of IL-17, IL-6, and TNF-α in mice colons. Data presented as mean ± standard deviation (SD). Statistical comparison was performed by one-way ANOVA with Holm–Šidák’s multiple comparison test. ^*^p < 0.05, ^**^p < 0.01, ^***^p < 0.001, and ^****^p < 0.0001.

As shown in Figure 3, compared with the control group, the levels of IL-17, IL-6, and TNF-α in serum and colon tissues of the DSS group were significantly increased. Compared with the DSS group, the levels of IL-17, IL-6, and TNF-α in colon tissues and IL-6 in serum of the Wc1982 group were decreased (*p* < 0.05). In addition, serum LPS in the DSS group was higher than that in the control group; however, it was significantly decreased in the Wc1982 group (*p* < 0.05).

### 3.4 *W. confusa* Wc1982 modified the gut microbiota composition

**Figure 4:**
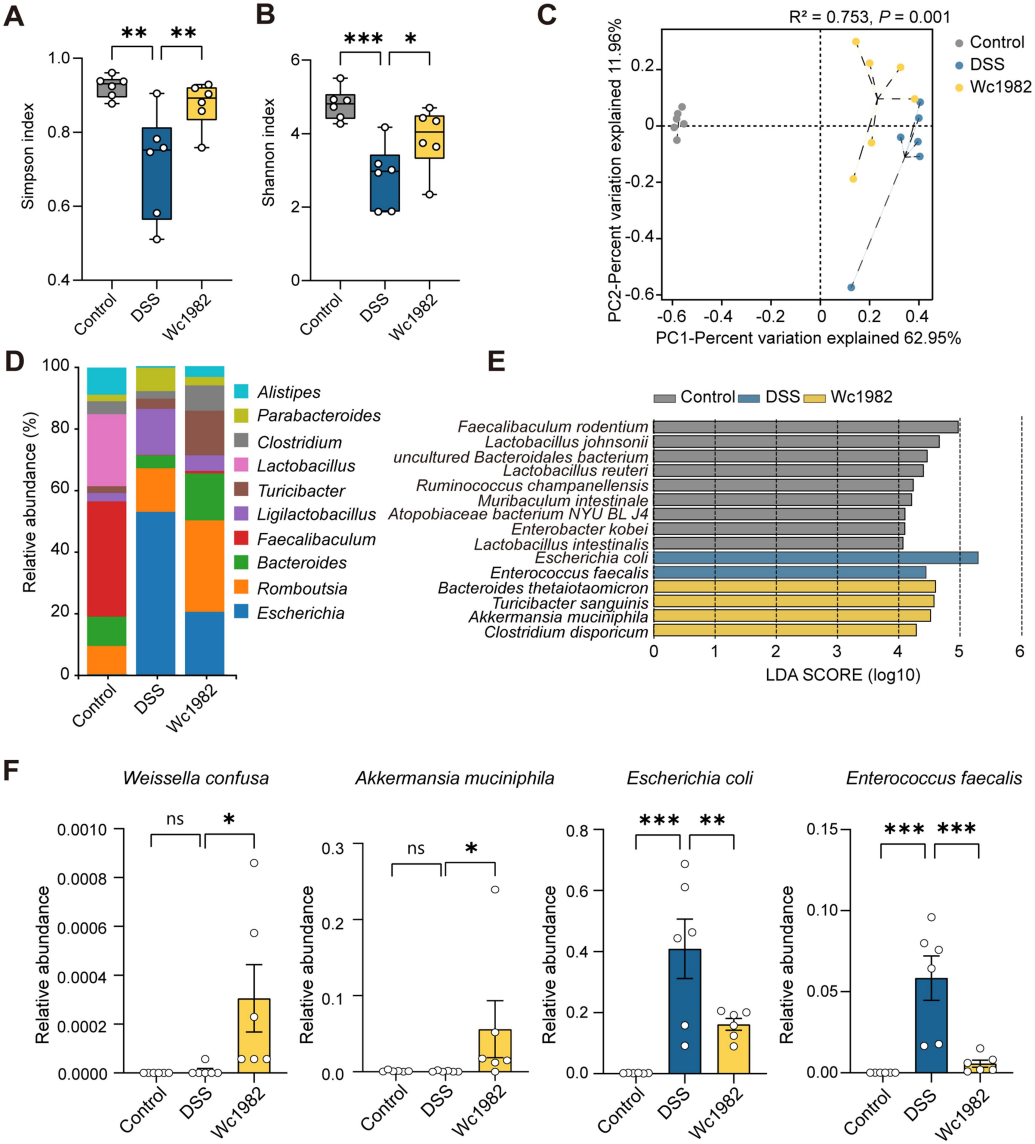
Effect of *W. confusa* Wc1982 on the composition and abundance of gut microbiota in mice with DSS-induced colitis. **(A,B)** Boxplots of the Simpson and Shannon indexes of α-diversity index among the three groups. **(C)** PCoA of β-diversity was performed based on the Pearson distance method. **(D)** Stacked bar chart of the microbial compositional profiling at the genus level (top 10). **(E)** Overrepresented bacterial taxa among groups determined by linear discriminant analysis (LDA). **(F)** Relative abundance of *W. confusa*, *A. muciniphila*, *E. coli*, and *E. faecalis*. Data presented as mean ± standard deviation (SD). Statistical comparison was performed by one-way ANOVA with Holm–Šidák’s multiple comparison test. ^*^p < 0.05, ^**^p < 0.01, and ^***^p < 0.001.

Cecal contents were analyzed by high throughput 16S rRNA amplicon sequencing to assess the composition of the gut microbiota. Compared with the control and Wc1982 groups, the Shannon and Simpson indexes in the DSS group were significantly reduced (*p* < 0.05, Figures 4A,B), but the ACE and Chao1 indexes of the DSS and Wc1982 groups were comparable (*p* < 0.05, Supplementary Figure S2). PCoA showed that there were significant differences in the composition of gut microbiota among the three groups (*p* < 0.05, Figure 4C). The top 10 abundant genera among the control, DSS, and Wc1982 groups were shown in Figure 4D. The relative abundance of *Faecalibaculum* was highest in the control group, *Escherichia* was the dominant genus in the DSS group, while *Romboutsia* was the dominant genus in the Wc1982 group.

LEfSe identified overrepresentation of three groups at the species level: *Bacteroides thetaiotaomicron* and *Akkermansia muciniphila* were enriched in the Wc1982 group, *E. coli* and *Enterococcus faecalis* were enriched in the DSS group, and *Lactobacillus johnsoni* and *Lactobacillus reuteri* were enriched in the control group (Figure 4E). The relative abundance of *W. confusa* and *A. muciniphila* in the Wc1982 group was higher than that in the DSS group, while the relative abundance of *E. coli* and *E. faecalis* in the Wc1982 group was lower than those in the DSS group (*p* < 0.05, Figure 4F).

### 3.5 Correlation of the gut microbiota with inflammatory indicators

**Figure 5:**
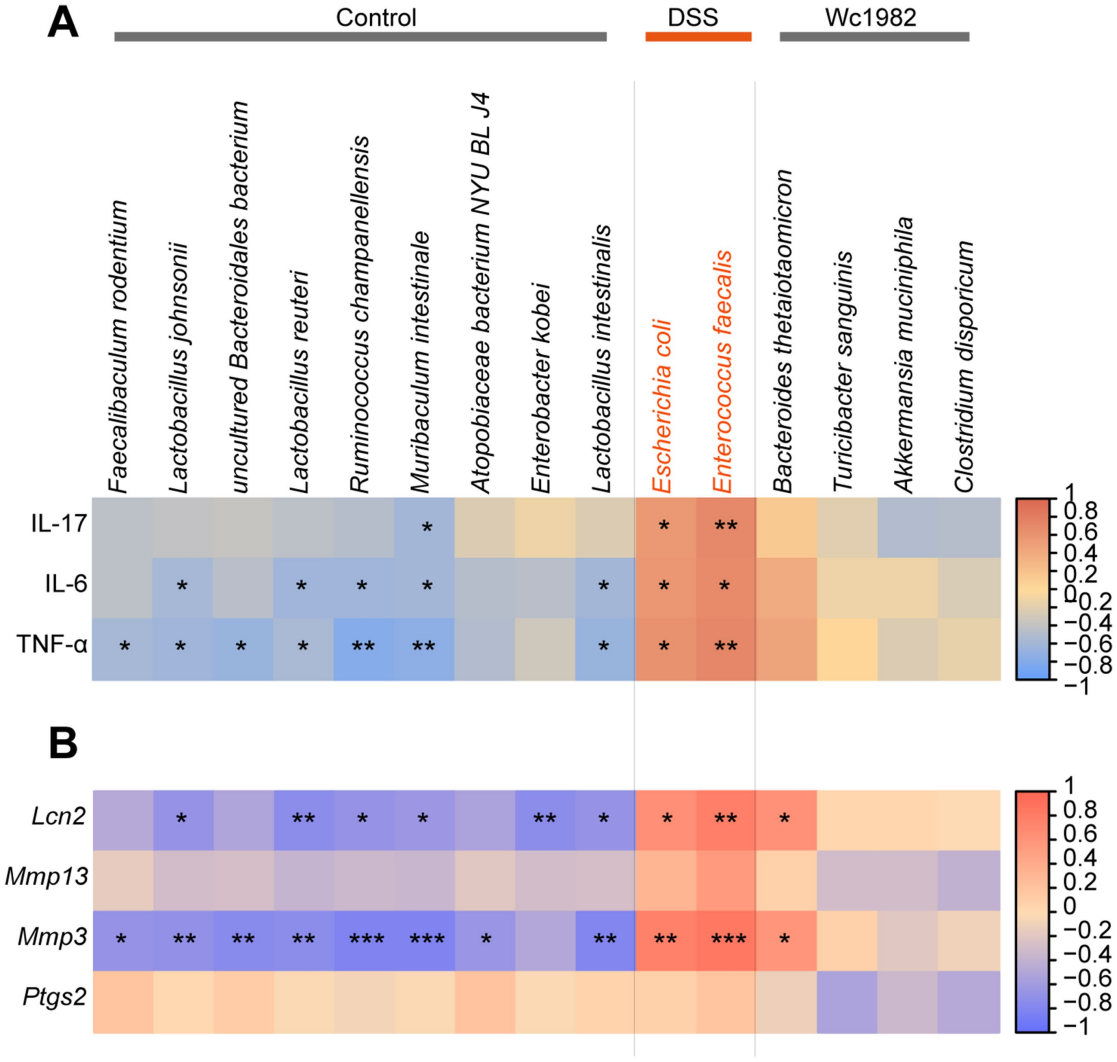
Interaction between gut microbiota with cytokines or core DEGs in the IL-17 signaling pathway. **(A)** Correlation of significantly different species of LEfSe with cytokines. **(B)** Correlation of significantly different species of LEfSe with core DEGs. Significant correlations are marked by ^*^FDR <0.05, ^**^FDR <0.01, and ^***^FDR <0.001.

To investigate the correlation between gut microbiota and inflammation indicators, we performed a Spearman rank correlation analysis and visualized the correlation between the relative abundance of significantly different species and the levels of colon cytokines or the expressions of four DEGs related to the IL-17 signaling pathway in Figures 5A,B, respectively. *E. coli* and *E. faecalis* showed positive correlations with colon cytokines, including IL-17, IL-6, and TNF-α, as well as with *Lcn2* and *Mmp3* genes. However, *Muribaculum* *intestinale* showed opposite trends of association with these colon cytokines and genes. *Lactobacillus johnsonii*, *Lactobacillus reuteri*, *Ruminococcus champanellensis*, and *Lactobacillus intestinalis* were the main microbiota negatively correlated with IL-6, TNF-α, *Lcn2*, and *Mmp3* genes.

## 4 Discussion

Studies have shown that various *W. confusa* strains reduce intestinal inflammation (Ahmed et al., 2022; Sujaya et al., 2023). In this study we used a *W. confusa* strain, Wc1982, isolated from a healthy human donor, which showed similar anti-inflammatory effect, suggesting that it is a general property of *W. confusa* Wc1982 exerted protective effects in DSS-induced colitis in mice by reducing the DAI and pathological scores, suppressing colon shortening, and alleviating epithelial injury and inflammatory cell infiltration.

Notably, the protective role of Wc1982 was closely associated with the regulation of the IL-17 signaling pathway, as evidenced by significant downregulation of key genes (*Lcn2*, *Mmp3*, *Mmp13*, and *Ptgs2*) in colon tissues following intervention. Lcn2 is known to play an inflammatory role in colitis through mediating intestinal epithelial cell pyroptosis (Yang et al., 2024). In our study, the Wc1982 group had reduced *Lcn2* expression with restored mucosal integrity and reduced histopathological damage. Mmp3 and Mmp13, are matrix metalloproteinases (MMPs) that are critical for inflammatory tissue remodeling and promote intestinal fibrosis and lumen narrowing (De Bruyn et al., 2016). In our study, in the DSS group, we found elevated *Mmp3/13* levels accompanied by colon shortening, aligning with their established role in fibrosis-driven structural damage (De Bruyn et al., 2016), while Wc1982 intervention reduced *Mmp3/13* expression and partially restored colon length, suggesting that Wc1982 alleviated fibrosis-driven structural damage. Inhibition of *Ptgs2* using inhibitors has been shown to alleviate colitis (El Miedany et al., 2006). The Wc1982 group had reduced *Ptgs2* expression, suggesting microbiota (*W. confusa*)-driven anti-inflammation.

Gut microbiota dysbiosis, particularly the overgrowth of *E. coli* and *E. faecalis*, exacerbates colitis through IL-17-driven inflammatory cascades (Paroni et al., 2023). This dysbiosis disrupts the natural composition of commensal microbiota, as evidenced by the depletion of species commonly present in healthy hosts (Zhang et al., 2023; Ben David et al., 2015; Miyake et al., 2020; Lee et al., 2025; Fujisawa et al., 1990). This was also reflected in animal models, with *Muribaculum intestinale*, *Ruminococcus champanellensis*, and *Lactobacillus* spp. (*L. johnsonii*, *L. intestinalis*, and *L. reuteri*) reduced in DSS-induced groups. These symbionts were exclusively enriched in the control group in our study, where they exhibited strong negative correlations with pro-inflammatory cytokines (IL-6, TNF-α) and inflammation-related genes (*Lcn2*, *Mmp3*), suggesting their protective role in mitigating colitis severity. Conversely, pathological organisms such as adherent-invasive *E. coli* (AIEC) and *E. faecalis* dominate in dysbiotic conditions. AIEC activates pathogenic Th17 (pTh17) cells and induces dendritic cells (DCs) to secrete IL-23, thereby promoting IL-17 production (Paroni et al., 2023). This cytokine subsequently upregulates *Lcn2* mRNA expression (Chiricozzi et al., 2016). *E. faecalis* compromises tight junctions via lipoteichoic acid, facilitating bacterial translocation and systemic IL-6/IL-17 release (Tian et al., 2023). In both cases, IL-17 signaling is amplified, which drives extracellular matrix degradation (via upregulation of *Mmp3/Mmp13*) that culminates in colonic atrophy (De Bruyn et al., 2016). In our study, Wc1982 intervention appeared to disrupt this inflammatory cycle through targeting microbiota and host responses. The reduction in *E. coli*/*E. faecalis* abundance was paralleled by attenuated IL-17 levels, which may mechanistically contribute to the downregulation of *Lcn2* and *Mmp3/13.*

Beyond suppressing *E. coli* and *E. faecalis*, Wc1982 intervention further reshaped the structure of gut microbiota by selectively enriching mucin-utilizing symbionts, particularly *A. muciniphila*. Short-chain fatty acids produced by *W. confusa* (Elshaghabee et al., 2020) may further enhance mucosal health by stimulating goblet cells to produce mucin (MUC2), the primary energy source for *A. muciniphila* (Sanjiwani et al., 2022). Furthermore, *W. confusa* may mitigate oxidative stress (Wang et al., 2022; Zhao et al., 2021) and lower pH (Tuccillo et al., 2022), creating an unfavorable niche for *Enterobacteriaceae* (Moreira De Gouveia et al., 2024; Yin et al., 2025) that compete with *A. muciniphila*. However, the causality between microbial modulation and molecular changes, as well as the exact interplay between *A. muciniphila* and *W. confusa*, requires further validation.

Collectively, Wc1982 has the potential as a next-generation probiotic for IBD intervention. We showed that Wc1982 reduced colon shortening by 17.59% (vs. DSS group, *p* < 0.05) and decreased DAI by 18% (vs. DSS group, *p* < 0.05)—effects comparable to strains like *B. bifidum* [colon shortening decreased by 11.76–25.97% (Cui et al., 2022, Feng et al., 2022, Kang et al., 2022)] and *L. plantarum* [DAI decreased by 19.67–30.38% (Qin et al., 2022, Kim et al., 2020)]. Further, Wc1982 may complement *Bifidobacterium* and *Lactobacillus* in alleviating colitis (Kim et al., 2020; Vanderpool et al., 2008). *Bifidobacterium* and *Lactobacillus* enhance IL-10 expression and inhibit TNF-α, IL-6, and NF-κB (Kim et al., 2020, Vanderpool et al., 2008), whereas Wc1982 mainly suppressed IL-17 expression. There is potential combining them as probiotics in mixed formulations to enhance their effect.

However, our study design had several limitations: (1) model specificity: the experimental results were derived solely from a DSS-induced colitis mouse model, which does not fully replicate human IBD pathophysiology. Further validation of the efficacy of Wc1982 in complementary IBD models—such as trinitrobenzenesulfonic acid-induced colitis and IL-10^−/−^ mice would be useful. (2) Microbiome confounders: DSS-induced gut microbiota disruption may obscure the direct mechanistic effects of Wc1982 on microbial modulation. Validation through germ-free models or antibiotic pretreatment systems is critical to disentangle these interactions. (3) Translational constraints: current data lack clinical validation, limiting conclusions on the efficacy of Wc1982 and its safety in humans. Clinical trials of Wc1982 are needed to evaluate human safety, dose–response relationships, and microbiome-immune interactions.

## 5 Conclusion

This study demonstrated that *W. confusa* Wc1982 had a protective effect against DSS-induced mouse colitis, by restructuring gut microbiota composition, reducing serum LPS levels, inhibiting the expression of colonic pro-inflammatory cytokines IL-17, IL-6, and TNF-α, and regulating the expressions of inflammation-related genes. However, the specific mechanism by which *W. confusa* Wc1982 interacts with the host to exert therapeutic effects requires further investigation.

## Data Availability

The datasets presented in this study can be found in online repositories. The names of the repository/repositories and accession number(s) can be found at: https://www.ncbi.nlm.nih.gov/, PRJNA1201878, https://www.ncbi.nlm.nih.gov/, PRJNA1178434.

## References

[ref1] Ahmed S. Singh S. Singh V. Roberts K. D. Zaidi A. Rodriguez-Palacios A. (2022). The *Weissella* genus: clinically treatable bacteria with antimicrobial/probiotic effects on inflammation and cancer. Microorganisms 10:2427. doi: 10.3390/microorganisms10122427, PMID: 36557680 PMC9788376

[ref2] Ben David Y. Dassa B. Borovok I. Lamed R. Koropatkin N. M. Martens E. C.. (2015). Ruminococcal cellulosome systems from rumen to human. Environ. Microbiol. 17, 3407–3426. doi: 10.1111/1462-2920.12868 25845888

[ref3] Bokulich N. A. Dillon M. R. Zhang Y. Rideout J. R. Bolyen E. Li H.. (2018). Q2-longitudinal: longitudinal and paired-sample analyses of microbiome data. mSystems 3:e00219. doi: 10.1128/mSystems.00219-18, PMID: 30505944 PMC6247016

[ref4] Chiricozzi A. Suárez-Fariñas M. Fuentes-Duculan J. Cueto I. Li K. Tian S.. (2016). Increased expression of interleukin-17 pathway genes in nonlesional skin of moderate-to-severe psoriasis vulgaris. Br. J. Dermatol. 174, 136–145. doi: 10.1111/bjd.14034, PMID: 26189551 PMC4720589

[ref5] Cui Q. Y. Tian X. Y. Liang X. Zhang Z. Wang R. Zhou Y.. (2022). *Bifidobacterium bifidum* relieved DSS-induced colitis in mice potentially by activating the aryl hydrocarbon receptor. Food Funct. 13, 5115–5123. doi: 10.1039/d1fo04219j, PMID: 35416187

[ref6] De Bruyn M. Vandooren J. Ugarte-Berzal E. Arijs I. Vermeire S. Opdenakker G. (2016). The molecular biology of matrix metalloproteinases and tissue inhibitors of metalloproteinases in inflammatory bowel diseases. Crit. Rev. Biochem. Mol. Biol. 51, 295–358. doi: 10.1080/10409238.2016.1199535, PMID: 27362691

[ref7] Dey D. K. Kang S. C. (2020). *Weissella confusa* DD_A7 pre-treatment to zebrafish larvae ameliorates the inflammation response against *Escherichia coli* O157: H7. Microbiol. Res. 237:126489. doi: 10.1016/j.micres.2020.126489, PMID: 32464536

[ref8] Dey D. K. Khan I. Kang S. C. (2019). Anti-bacterial susceptibility profiling of *Weissella confusa* DD_A7 against the multidrug-resistant ESBL-positive *E. coli*. Microb. Pathog. 128, 119–130. doi: 10.1016/j.micpath.2018.12.048, PMID: 30597254

[ref9] Edgar R. C. Haas B. J. Clemente J. C. Quince C. Knight R. (2011). Uchime improves sensitivity and speed of chimera detection. Bioinformatics 27, 2194–2200. doi: 10.1093/bioinformatics/btr381, PMID: 21700674 PMC3150044

[ref10] El Miedany Y. Youssef S. Ahmed I. El Gaafary M. (2006). The gastrointestinal safety and effect on disease activity of etoricoxib, a selective COX-2 inhibitor in inflammatory bowel diseases. Am. J. Gastroenterol. 101, 311–317. doi: 10.1111/j.1572-0241.2006.00384.x, PMID: 16454836

[ref11] Elshaghabee F. M. F. Ghadimi D. Habermann D. De Vrese M. Bockelmann W. Kaatsch H. J.. (2020). Effect of oral administration of *Weissella confusa* on fecal and plasma ethanol concentrations, lipids and glucose metabolism in wistar rats fed high fructose and fat diet. Hepat. Med. 12, 93–106. doi: 10.2147/hmer.S254195, PMID: 32617026 PMC7326399

[ref12] Feng C. Zhang W. Zhang T. He Q. Kwok L. Y. Tan Y.. (2022). Heat-killed *Bifidobacterium bifidum* b1628 may alleviate dextran sulfate sodium-induced colitis in mice, and the anti-inflammatory effect is associated with gut microbiota modulation. Nutrients 14:5233. doi: 10.3390/nu14245233, PMID: 36558391 PMC9784753

[ref13] Fujisawa T. Itoh K. Benno Y. Mitsuoka T. (1990). *Lactobacillus intestinalis* (ex hemme 1974) sp. Nov., nom. Rev., isolated from the intestines of mice and rats. Int. J. Syst. Bacteriol. 40, 302–304. doi: 10.1099/00207713-40-3-302, PMID: 2397198

[ref14] Ghadimi D. Folster-Holst R. Rocken C. Kaatsch H. J. Ebsen M. Tournebize R.. (2023). Endogenous ethanol-producing bacteria interference in pathogen-host crosstalk. Endocr. Metab. Immune Disord. Drug Targets 23, 1430–1441. doi: 10.2174/1871530323666230330111355, PMID: 36998141

[ref15] Goodoory V. C. Khasawneh M. Black C. J. Quigley E. M. M. Moayyedi P. Ford A. C. (2023). Efficacy of probiotics in irritable bowel syndrome: systematic review and meta-analysis. Gastroenterology 165, 1206–1218. doi: 10.1053/j.gastro.2023.07.018, PMID: 37541528

[ref16] Guan Q. (2019). A comprehensive review and update on the pathogenesis of inflammatory bowel disease. J Immunol Res 2019:7247238. doi: 10.1155/2019/7247238, PMID: 31886308 PMC6914932

[ref17] Hamed S. A. Mohan A. Navaneetha Krishnan S. Wang A. Drikic M. Prince N. L.. (2023). Butyrate reduces adherent-invasive *E. coli*-evoked disruption of epithelial mitochondrial morphology and barrier function: involvement of free fatty acid receptor 3. Gut Microbes 15:2281011. doi: 10.1080/19490976.2023.2281011, PMID: 38078655 PMC10730202

[ref18] Haneishi Y. Furuya Y. Hasegawa M. Picarelli A. Rossi M. Miyamoto J. (2023). Inflammatory bowel diseases and gut microbiota. Int. J. Mol. Sci. 24:3817. doi: 10.3390/ijms24043817, PMID: 36835245 PMC9958622

[ref19] Haque M. Kaminsky L. Abdulqadir R. Engers J. Kovtunov E. Rawat M.. (2024). *Lactobacillus acidophilus* inhibits the TNF-α-induced increase in intestinal epithelial tight junction permeability via a TLR-2 and PI3K-dependent inhibition of NF-κB activation. Front. Immunol. 15:1348010. doi: 10.3389/fimmu.2024.1348010, PMID: 39081324 PMC11286488

[ref20] Kang S. Lin Z. Xu Y. Park M. Ji G. E. Johnston T. V.. (2022). A recombinant *Bifidobacterium bifidum* BGN4 strain expressing the streptococcal superoxide dismutase gene ameliorates inflammatory bowel disease. Microb. Cell Fact. 21:113. doi: 10.1186/s12934-022-01840-2, PMID: 35672695 PMC9172062

[ref21] Kim D. H. Kim S. Ahn J. B. Kim J. H. Ma H. W. Seo D. H.. (2020). *Lactobacillus plantarum* CBT LP3 ameliorates colitis via modulating t cells in mice. Int. J. Med. Microbiol. 310:151391. doi: 10.1016/j.ijmm.2020.151391, PMID: 32007342

[ref22] Klindworth A. Pruesse E. Schweer T. Peplies J. Quast C. Horn M.. (2013). Evaluation of general 16s ribosomal RNA gene PCR primers for classical and next-generation sequencing-based diversity studies. Nucleic Acids Res. 41:e1. doi: 10.1093/nar/gks808, PMID: 22933715 PMC3592464

[ref23] Lee A. H. Rodriguez Jimenez D. M. Meisel M. (2025). *Limosilactobacillus reuteri*—a probiotic gut commensal with contextual impact on immunity. Gut Microbes 17:2451088. doi: 10.1080/19490976.2025.2451088 39825615 PMC12716054

[ref24] Li Q. Li Y. Song J. Xu H. Xu J. Zhu Y.. (2014). High-accuracy *de novo* assembly and SNP detection of chloroplast genomes using a SMRT circular consensus sequencing strategy. New Phytol. 204, 1041–1049. doi: 10.1111/nph.12966, PMID: 25103547

[ref25] Li Q. Sun X. Yu K. Lv J. Miao C. Yang J.. (2022). *Enterobacter ludwigii* protects DSS-induced colitis through choline-mediated immune tolerance. Cell Rep. 40:111308. doi: 10.1016/j.celrep.2022.111308, PMID: 36044853

[ref26] Li D. Zand M. S. Dye T. D. Goniewicz M. L. Rahman I. Xie Z. (2022). An evaluation of RNA-seq differential analysis methods. PLoS One 17:e0264246. doi: 10.1371/journal.pone.0264246, PMID: 36112652 PMC9480998

[ref27] Liu L. Du Y. Du Y. Yan W. Li Y. Cui K.. (2023). Exopolysaccharide from *Weissella confusa* J4-1 inhibits colorectal cancer via induction of cell cycle arrest. Int. J. Biol. Macromol. 253:127625. doi: 10.1016/j.ijbiomac.2023.127625, PMID: 37884233

[ref28] Martyniak A. Medyńska-Przęczek A. Wędrychowicz A. Skoczeń S. Tomasik P. J. (2021). Prebiotics, probiotics, synbiotics, paraprobiotics and postbiotic compounds in IBD. Biomol. Ther. 11:1903. doi: 10.3390/biom11121903, PMID: 34944546 PMC8699341

[ref29] Mei Y. Wang Z. Zhang Y. Wan T. Xue J. He W.. (2019). FA-97, a new synthetic caffeic acid phenethyl ester derivative, ameliorates DSS-induced colitis against oxidative stress by activating Nrf2/HO-1 pathway. Front. Immunol. 10:2969. doi: 10.3389/fimmu.2019.02969, PMID: 31969881 PMC6960141

[ref30] Miyake S. Ding Y. Soh M. Low A. Seedorf H. (2020). *Muribaculum gordoncarteri* sp. Nov., an anaerobic bacterium from the faeces of C57BL/6J mice. Int. J. Syst. Evol. Microbiol. 70, 4725–4729. doi: 10.1099/ijsem.0.004338, PMID: 32687462

[ref31] Moraes L. C. Lang P. M. Arcanjo R. A. Rampelotto P. H. Fatturi-Parolo C. C. Ferreira M. B. C.. (2020). Microbial ecology and predicted metabolic pathways in various oral environments from patients with acute endodontic infections. Int. Endod. J. 53, 1603–1617. doi: 10.1111/iej.13389, PMID: 33448446

[ref32] Moreira De Gouveia M. I. Bernalier-Donadille A. Jubelin G. (2024). Enterobacteriaceae in the human gut: dynamics and ecological roles in health and disease. Biology 13:142. doi: 10.3390/biology13030142, PMID: 38534413 PMC10967970

[ref33] Nishida A. Inoue R. Inatomi O. Bamba S. Naito Y. Andoh A. (2018). Gut microbiota in the pathogenesis of inflammatory bowel disease. Clin. J. Gastroenterol. 11, 1–10. doi: 10.1007/s12328-017-0813-5, PMID: 29285689

[ref34] Paroni M. Leccese G. Ranzani V. Moschetti G. Chiara M. Perillo F.. (2023). An intestinal Th17 subset is associated with inflammation in Crohn’s disease and activated by adherent-invasive *Escherichia coli*. J. Crohns Colitis 17, 1988–2001. doi: 10.1093/ecco-jcc/jjad119, PMID: 37462681 PMC10798865

[ref35] Peyrin-Biroulet L. Panes J. Sandborn W. J. Vermeire S. Danese S. Feagan B. G.. (2016). Defining disease severity in inflammatory bowel diseases: current and future directions. Clin. Gastroenterol. Hepatol. 14, 348–354.e17. doi: 10.1016/j.cgh.2015.06.001, PMID: 26071941

[ref36] Qian K. Chen S. Wang J. Sheng K. Wang Y. Zhang M. (2022). A β-n-acetylhexosaminidase Amuc_2109 from *Akkermansia muciniphila* protects against dextran sulfate sodium-induced colitis in mice by enhancing intestinal barrier and modulating gut microbiota. Food Funct. 13, 2216–2227. doi: 10.1039/d1fo04094d, PMID: 35133390

[ref37] Qin S. Huang Z. Wang Y. Pei L. Shen Y. (2022). Probiotic potential of *Lactobacillus* isolated from horses and its therapeutic effect on DSS-induced colitis in mice. Microb. Pathog. 165:105216. doi: 10.1016/j.micpath.2021.105216, PMID: 34600098

[ref38] Quast C. Pruesse E. Yilmaz P. Gerken J. Schweer T. Yarza P.. (2013). The SILVA ribosomal RNA gene database project: improved data processing and web-based tools. Nucleic Acids Res. 41, D590–D596. doi: 10.1093/nar/gks1219, PMID: 23193283 PMC3531112

[ref39] Rognes T. Flouri T. Nichols B. Quince C. Mahé F. (2016). VSEARCH: a versatile open source tool for metagenomics. PeerJ 4:e2584. doi: 10.7717/peerj.2584, PMID: 27781170 PMC5075697

[ref40] Sanjiwani M. I. D. Aryadi I. P. H. Semadi I. M. S. (2022). Review of literature on *Akkermansia muciniphila* and its possible role in the etiopathogenesis and therapy of type 2 diabetes mellitus. J. ASEAN Fed Endocr. Soc. 37, 69–74. doi: 10.15605/jafes.037.01.13, PMID: 35800592 PMC9242659

[ref41] Sann H. Erichsen J. Hessmann M. Pahl A. Hoffmeyer A. (2013). Efficacy of drugs used in the treatment of IBD and combinations thereof in acute DSS-induced colitis in mice. Life Sci. 92, 708–718. doi: 10.1016/j.lfs.2013.01.028, PMID: 23399699

[ref42] Selvamani S. Mehta V. Ali El Enshasy H. Thevarajoo S. El Adawi H. Zeini I.. (2022). Efficacy of probiotics-based interventions as therapy for inflammatory bowel disease: a recent update. Saudi J. Biol. Sci. 29, 3546–3567. doi: 10.1016/j.sjbs.2022.02.044, PMID: 35844369 PMC9280206

[ref43] Sujaya I. N. Dharmika I. Suwardana G. N. R. Mariadi I. K. Arijana I. Winaya I. B. O.. (2023). *Weissella confusa* F213 ameliorated inflammation and maintained intestinal mucosa integrity in chemically induced colitis rats. BMC. Res. Notes 16:178. doi: 10.1186/s13104-023-06456-2, PMID: 37608379 PMC10463849

[ref44] Tian Z. Khan A. I. Rehman A. U. Deng T. Ma C. Wang L. (2023). Virulence factors and mechanisms of paediatric pneumonia caused by *Enterococcus faecalis*. Gut Pathog. 15:2. doi: 10.1186/s13099-022-00522-z, PMID: 36624474 PMC9830894

[ref45] Tian M. Wang H. Li Y. Wang J. Ren D. Lin K. (2025). A comprehensive safety assessment of a novel starter *Weissella confusa* M1 combining with whole-genome sequencing. Food Res. Int. 202:115748. doi: 10.1016/j.foodres.2025.115748, PMID: 39967109

[ref46] Tuccillo F. Wang Y. Edelmann M. Lampi A. M. Coda R. Katina K. (2022). Fermentation conditions affect the synthesis of volatile compounds, dextran, and organic acids by *Weissella confusa* A16 in faba bean protein concentrate. Foods 11:3579. doi: 10.3390/foods11223579 36429171 PMC9689515

[ref47] Vallejos O. P. Bueno S. M. Kalergis A. M. (2025). Probiotics in inflammatory bowel disease: microbial modulation and therapeutic prospects. Trends Mol. Med. 14, S1471–S4914. doi: 10.1016/j.molmed.2024.12.005, PMID: 39814640

[ref48] Vanderpool C. Yan F. Polk D. B. (2008). Mechanisms of probiotic action: implications for therapeutic applications in inflammatory bowel diseases. Inflamm. Bowel Dis. 14, 1585–1596. doi: 10.1002/ibd.20525, PMID: 18623173

[ref49] Walker J. E. Oliver J. C. Stewart A. M. Beh S. T. Arce R. A. Glass M. J.. (2023). Measuring up: a comparison of Tapestation 4200 and Bioanalyzer 2100 as measurement tools for RNA quality in postmortem human brain samples. Int. J. Mol. Sci. 24:13795. doi: 10.3390/ijms241813795, PMID: 37762097 PMC10531353

[ref50] Wang W. Li S. Heng X. Chu W. (2022). *Weissella confusa* CGMCC 19,308 strain protects against oxidative stress, increases lifespan, and bacterial disease resistance in *Caenorhabditis elegans*. Probiotics Antimicrob. Proteins 14, 121–129. doi: 10.1007/s12602-021-09799-z, PMID: 34037943

[ref51] Wang L. Tang L. Feng Y. Zhao S. Han M. Zhang C.. (2020). A purified membrane protein from *Akkermansia muciniphila* or the pasteurised bacterium blunts colitis associated tumourigenesis by modulation of CD8^+^ T cells in mice. Gut 69, 1988–1997. doi: 10.1136/gutjnl-2019-320105, PMID: 32169907 PMC7569398

[ref52] Wang L. Xie H. Xu L. Liao Q. Wan S. Yu Z.. (2017). Rsj16 protects against DSS-induced colitis by inhibiting the PPAR-α signaling pathway. Theranostics 7, 3446–3460. doi: 10.7150/thno.20359, PMID: 28912887 PMC5596435

[ref53] Wang H. Zhang X. Kou X. Zhai Z. Hao Y. (2023). A ropy exopolysaccharide-producing strain *Bifidobacterium pseudocatenulatum* Bi-OTA128 alleviates dextran sulfate sodium-induced colitis in mice. Nutrients 15:4993. doi: 10.3390/nu15234993, PMID: 38068850 PMC10707796

[ref54] Wong W. Y. Chan B. D. Sham T. T. Lee M. M. Chan C. O. Chau C. T.. (2022). *Lactobacillus casei* strain shirota ameliorates dextran sulfate sodium-induced colitis in mice by increasing taurine-conjugated bile acids and inhibiting NF-κB signaling via stabilization of IκBα. Front. Nutr. 9:816836. doi: 10.3389/fnut.2022.816836, PMID: 35529468 PMC9069136

[ref55] Yang Y. Li S. Liu K. Zhang Y. Zhu F. Ben T.. (2024). Lipocalin-2-mediated intestinal epithelial cells pyroptosis via NF-κB/NLRP3/GSDMD signaling axis adversely affects inflammation in colitis. Biochim. Biophys. Acta 1870:167279. doi: 10.1016/j.bbadis.2024.167279, PMID: 38844113

[ref56] Ye Y. Pang Z. Chen W. Ju S. Zhou C. (2015). The epidemiology and risk factors of inflammatory bowel disease. Int. J. Clin. Exp. Med. 8, 22529–22542, PMID: 26885239 PMC4730025

[ref57] Yin Q. Da Silva A. C. Zorrilla F. Almeida A. S. Patil K. R. Almeida A. (2025). Ecological dynamics of Enterobacteriaceae in the human gut microbiome across global populations. Nat. Microbiol. 10, 541–553. doi: 10.1038/s41564-024-01912-6, PMID: 39794474 PMC11790488

[ref58] Zhang Z. Zhao L. Wu J. Pan Y. Zhao G. Li Z.. (2023). The effects of *Lactobacillus johnsonii* on diseases and its potential applications. Microorganisms 11:2580. doi: 10.3390/microorganisms11102580, PMID: 37894238 PMC10609197

[ref59] Zhao D. Jiang J. Liu L. Wang S. Ping W. Ge J. (2021). Characterization of exopolysaccharides produced by *Weissella confusa* XG-3 and their potential biotechnological applications. Int. J. Biol. Macromol. 178, 306–315. doi: 10.1016/j.ijbiomac.2021.02.182, PMID: 33652047

