# Supplementary material for: Weissella confusa alleviates experimental colitis in mice by regulating inflammatory pathways and gut microbiota
Source: Front Microbiol. 2025 Apr 28;16:1574548. doi: 10.3389/fmicb.2025.1574548 (PMC12068860; doi:10.3389/fmicb.2025.1574548)
Supplement: Supplementary file 1 [file Supplementary_file_1.docx]

Supplementary Material

## Supplementary Figures

**
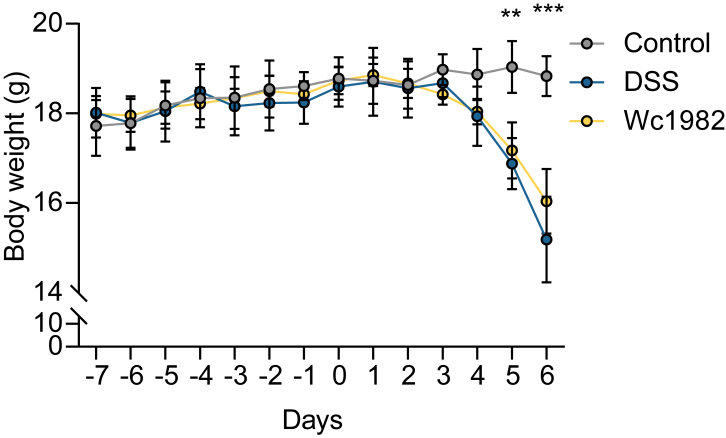
**

**Figure S1. Weight changes of three groups of mice from day -7 to 6.** Data presented as mean ± standard deviation (SD). Statistical comparison was performed by two-way ANOVA with Tukey's multiple comparison test. ** *P* < 0.01, *** *P* < 0.001.


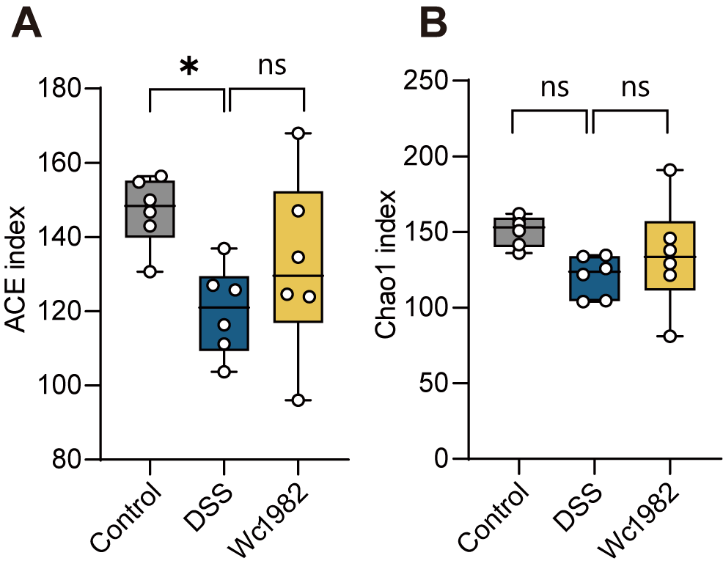


**Figure S2. Microbiota abundance alterations of different groups.** (A) ACE index. (B) Chao1 index. Data presented as mean ± standard deviation (SD). Statistical comparison was performed by one-way ANOVA with Holm-Šidák's multiple comparison test. * *P* < 0.05.
